# On the performance of uplink D2D-assisted backscatter employing short packet communication

**DOI:** 10.1371/journal.pone.0336406

**Published:** 2025-11-26

**Authors:** Si-Phu Le, Bui Vu Minh, Vu Quang Sy, Miroslav Voznak

**Affiliations:** 1 Faculty of Electrical Engineering and Computer Science, VSB-Technical University of Ostrava, Ostrava, Czechia; 2 Faculty of Engineering and Technology, Nguyen Tat Thanh University, Ho Chi Minh City, Vietnam; 3 Advanced Intelligent Technology Research Group, Faculty of Electrical and Electronics Engineering, Ton Duc Thang University, Ho Chi Minh City, Vietnam; Beijing Institute of Technology, CHINA

## Abstract

This paper examines the synergistic integration of uplink Device-to-Device (D2D), backscatter, and short-packet communication paradigms, highlighting their collective potential to revolutionize next-generation wireless systems. By enhancing spectral efficiency and supporting massive connectivity through diverse receiver techniques, this approach is undeniably transformative. Then, we analyze the approximation forms of average block error ratio (BLER) across three scenarios: selective combining - random selection (SC-RAN), selective combining - maximal ratio combining (SC-MRC), and full-maximal ratio combining (Full-MRC). Results indicate that the full-MRC scheme consistently outperforms the others in reducing BLER, particularly in low-latency scenarios. The findings serve as a foundation for making strategic design decisions about the system’s core operational parameters. Our numerical results strongly validate our analytical findings, clearly demonstrating that the full-MRC technique significantly outperforms others in improving BLER.

## 1 Introduction

Device-to-Device (D2D) communication represents a contemporary advancement in wireless technology, facilitating direct interaction between two or more devices without relying on conventional network infrastructure like base stations or access points [1,2]. This direct connection among user equipment (UE) is engineered to enhance network efficiency, support novel applications, and address the increasing demand for high data rates and low latency within mobile networks [3,4]. It is commonly used in scenarios where devices need to share information quickly and securely [5], such as file sharing, multiplayer gaming, location-based services, and IoT (Internet of Things) applications [6,7]. Moreover, in uplink D2D communication as discussed in [8,9], specific, the authors in [8] assertively introduced and examined an innovative hybrid cellular and bidirectional D2D transmission scheme. This approach strategically employs cooperative non-orthogonal multiple access (NOMA) to improve communication efficiency. As detailed in [9], a semi-centralized cooperative control method is proposed with precision, incorporating two distinct cooperative schemes based on D2D communication.

Beside, Backscatter communication (BackCom) is a wireless communication technique that enables devices to transmit data by reflecting or modulating existing ambient radio frequency (RF) signals, rather than generating their own RF signals [10]. In backscatter communication, a device acts as a passive transponder, absorbing and then modulating RF signals from a nearby transmitter to convey information [11]. This allows the device to communicate with other devices or a central hub without the need for its own RF transmitter, significantly reducing power consumption and extending battery life [12]. One of the key advantages of backscatter communication is its low power consumption, making it suitable for battery-operated devices and IoT applications where energy efficiency is critical [13]. It also allows for long-range communication with minimal infrastructure requirements, as devices can leverage existing RF signals for communication [14]. In addition, the necessity for enhancing spectral and energy efficiency is becoming increasingly critical for the development of future wireless networks. In this context, Backcom technology, in conjunction with reconfigurable intelligent surfaces (RISs), has recently emerged as a promising solution to improve the performance of wireless communications [15–17]. Recently, some advance research combining with BackCom have been also introduced in [18–21]. In [18], Manzoor Ahmed et.al have utilized NOMA in conjunction with BackCom as a promising strategy to improve energy efficiency, maximize sum rates, ensure security, and optimize resource allocation. The authors in [19] proposed a framework of cognitive ambient backscatter communication (C-AmBC) networks in the presence of an unlicensed eavesdropper to investigate the reliability and security of their proposed framework by invoking the outage probability (OP) and intercept probability (IP) expressions. A mutualistic cooperative BackCom network with hardware impairments (HIs) at all the active transceivers and a non-linear energy harvesting circuit at each IoT device has been studied in [20]. Finally, the authors in [21] demonstrated the performance of BackCom over the position-flexible fluid antenna system (FAS) technology to evaluate the quality of their proposed system in terms of OP and the delay outage rate (DOR) derivations.

Another aspect in new areas of research, short packet communication (SPC) addresses the critical need for ultra-reliable low-latency transmission (URLLC) in 5G/6G applications, where blocklengths of <1,000 bits prioritize rapid delivery over traditional channel coding gains, and involves the transmission of small data packets between devices [22–24]. Finite blocklength theory reveals a fundamental trade-off: reducing packet length from 1,000 to 100 bits increases outage probability by 35–60% at 15 dB SNR, necessitating physical-layer security (PLS) mechanisms like artificial noise injection to maintain confidentiality [25,26]. In [27], the SPC is investigated by adopting the average block error ratio (BLER) under impact of co-channel interference affecting on the relays in a cooperative system where one multiple antenna transmitter communicates with one single antenna receiver with assistance of multiple relay nodes is considered. The other aspect, the authors in [28] proposed and studied performance of RISs in multi-hop communication networks employing SPC by calculating the end-to-end BLER of the proposed framework.

Furthermore, previous works have explored the combination of individual pairs of these technologies. For instance, backscatter has been integrated with D2D to enable wireless-powered relaying and energy-efficient cooperative transmission [29,30]. Meanwhile, SPC has been applied to D2D to improve reliability and latency in interference-limited scenarios [31,32]. Similarly, the combination of backscatter and SPC has been shown to enhance low-power communication efficiency while supporting hybrid operation between long and short packets [33,34]. These studies confirm the benefits of each pairwise combination, but to the best of our knowledge, no prior work has simultaneously considered D2D, BackCom, and SPC. Motivated by these promising results, in this paper we jointly integrate all three technologies. Our study leverages (i) the self-sustainability and energy efficiency of BackCom, (ii) the flexibility and proximity gains of D2D, and (iii) the low latency and high reliability of SPC. This synergy enables the design of self-sustainable, reliable, and ultra-low-latency systems for future wireless networks, which constitutes the main contribution of this manuscript. In particular, recent works have highlighted the importance of jointly considering reliability, latency, and energy efficiency when designing IoT networks. For example, studies such as [35–37] analyze energy-efficient transmission and resource allocation schemes for URLLC and demonstrate that optimizing system parameters can substantially reduce energy consumption without sacrificing reliability. Similarly, [38–40] investigate energy–latency tradeoffs and lightweight transmission strategies tailored to resource-constrained IoT devices. Moreover, the recent work [41] explicitly shows that certain resource configurations can achieve up to 80× higher energy efficiency compared to less efficient ones, while maintaining competitive latency performance. In addition, recent studies have explored related frameworks that further highlight the potential of combining backscatter and SPC. For example, the work in [42] investigates short-packet backscatter-assisted wireless-powered relaying with NOMA, providing mode-selection and performance-estimation strategies to optimize reliability and resource allocation. Likewise, cooperative and distributed multi-user detection techniques, such as those discussed in [43], demonstrate how scalable detection methods can significantly enhance system performance in densely connected scenarios. These studies emphasize the importance of incorporating both cooperative reception and backscatter–SPC integration into future wireless system designs. Despite these advances, the uplink D2D, BackCom and SPC lies in their combined potential to enhance the capabilities of connected devices. Furthermore, these technologies are further enhanced by diversity techniques such as selective combining (SC) and maximal ratio combining (MRC), which play a crucial role in improving communication reliability and performance in challenging wireless environments. SC selects the best signal path for reception, while MRC combines multiple received signals in proportion to their signal-to-noise ratios to improve the overall signal quality [44]. These diversity techniques help combat signal fading, interference, and other impairments, thereby enhancing communication performance in challenging wireless environments. However, it is worth noting that while Full-MRC provides the highest reliability, it also entails significant computational and hardware complexity, which may limit its feasibility in resource-constrained IoT deployments [45–47]. In contrast, low-complexity schemes such as SC-MRC can provide a more practical balance between performance and implementation cost, making them particularly attractive for large-scale IoT networks. On the other hands, in scenarios where devices need to communicate directly with each other while conserving energy and minimizing latency, a combination of D2D communication for local data exchange, backscatter communication for energy-efficient communication, and short packet communication for quick data transmission can offer a comprehensive solution. By leveraging the strengths of each technology, connected devices can establish efficient and reliable communication networks that cater to a wide range of applications in the IoT, smart infrastructure, and wireless sensor networks. This motivates us to conduct this research by providing a rigorous mathematical analysis framework for evaluating the average BLER with the combination of D2D, BackCom and SPC in our proposed system. The main contributions of the paper are outlined as follows:

- We proposed the uplink D2D-assisted BackCom network employing SPC to represent essential building blocks for enabling seamless connectivity among connected devices. Their synergistic relationship opens up possibilities for creating robust, energy-efficient, and low-latency communication systems that drive innovation in the realm of wireless networking and IoT applications.
- We derived the approximation-form expressions for the average BLER by using the Gaussian-Chebyshev quadrature in three cases: SC-RAN, SC-MRC and Full-MRC. To get full insights, some key parameters have been examined to bring more comprehensive solutions.
- Finally, the Monte Carlo simulation is conducted to clarify the accuracy of mathematical analysis.

**Table 1:** Comparison of the uniqueness of our research to related articles.

Context	Multi-antenna	MRC	SC	SPC	BackCom	D2D
Paper [29]					✓	✓
Paper [30]					✓	✓
Paper [31]				✓		✓
Paper [36]	✓	✓	✓			✓
Paper [37]	✓	✓	✓			✓
Paper [32]				✓		✓
Paper [45]	✓	✓	✓	✓		
Paper [46]	✓	✓		✓		
Paper [33]				✓	✓	
Paper [34]				✓	✓	
Paper [47]					✓	
Paper [35]	✓	✓			✓	
This paper	✓	✓	✓	✓	✓	✓

**Organization:** The subsequent sections of this manuscript are organized as follows. Sect 2 delineates the system description and signal modeling. The channel statistics and preliminary of finite blocklength is described in Sect 3. The BLER expressions analysis are presented in Sect 4. The numerical evaluations are shown in Sect 5. Lastly, Sect 6 encapsulates the findings of this manuscript.

## 2 System model

### 2.1 System description

**Fig 1:**
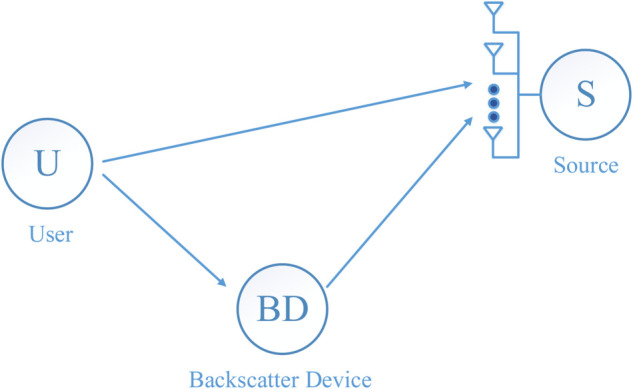
The system model of uplink communication with the existence of a backscatter device to reflect the user’s signal to the source.

In this Fig 1, the short-packet uplink transmission in symbiotic IoT networks from the user (U) to the source (S) is examined, wherein both U and the backscatter device (BD) are equipped with a single antenna, while S is furnished with multiple *M*-antennas. In this configuration, utilizing backscatter communication, the BD reflects the user’s signal to the source. In our proposed model, we assume that the channels among U–S, U–BD, and BD–S are independent block Rayleigh fading with perfect CSI. This assumption is widely adopted in the literature since it ensures analytical tractability and allows closed-form derivations of performance metrics such as BLER. Moreover, Rayleigh fading is often regarded as a worst-case scenario (i.e., Nakagami-*m* fading with *m* = 1 or Rician fading with *K* = 0), thereby providing a conservative benchmark compared to more favorable fading environments (e.g., Rician with *K* > 0 or Nakagami-*m* with *m* > 1). The use of Rayleigh fading is further justified by the fact that IoT devices typically operate in dense urban environments, where line-of-sight (LOS) links are frequently blocked by obstacles such as buildings and walls, leading to significant scattering and multipath propagation [48,49]. This channel model effectively captures the stochastic nature of amplitude variations in such scenarios.

**Table 2:** Notations of main parameters.

Symbol	Notation
*x*	Signal at User
*c*	Backscatter signal
*β*	The reflection coefficient
$P_{\text{U}}$	Transmit power at User
*h*_1_	Channel gain from *U* to *S*
*h*_2_	Channel gain from *U* to *BD*
*h*_3_	Channel gain from *BD* to *S*
$\lambda_{1}$	Mean of $|h_{1}{|}^{2}$
$\lambda_{2}$	Mean of $|h_{2}{|}^{2}$
$\lambda_{3}$	Mean of $|h_{3}{|}^{2}$
*L*	Packet length
*N*	The number of the information bits
χ	Path loss exponent
$f_{X}\left(\cdot \right)$	Probability density function (PDF) of *X*
$F_{X}\left(\cdot \right)$	Cumulative distribution function (CDF) of *X*
𝔼{·}	Expectation operator
|·|	The absolute value of a complex number
Γ(·)	Gamma function
γ(·,·)	Lower incomplete Gamma function
$K_{v}\left(\cdot \right)$	Bessel function of the second kind with $v^{\text{th}}$ order
$G_{p,q}^{m,n}\left(.\right)$	Meijer G-function

### 2.2 Signal model

Based on Fig 1, the received signal at S includes two parts: 1) signal directly *x*(*t*) satisfying $𝔼\{|x(t){|}^{2}\}=1$ from U over channel *h*_1_; and 2) reflecting signal *c*(*t*) satisfying $𝔼\{|c(t){|}^{2}\}=1$ from BD over cascade channel $h_{2}h_{3}$, which is a product of the channels from U to BD *h*_2_ and from BD to S *h*_3_. Therefore, the received signal at S can be expressed as:

$$y_{\text{S}}=\sqrt{P_{\text{U}}}h_{\text{1}}x\left(t\right)+\sqrt{\beta P_{\text{U}}}h_{\text{2}}h_{\text{3}}x\left(t\right)c(t)+n_{\text{S}},$$ (1)

where $n_{S}$ is the Additive Gaussian White Noise (AGWN) with zero mean and variance $N_{0}$, β>0 is a reflection coefficient used to normalize *c*(*t*), $P_{U}$ is the user transmit power. In symbiotic radio communication, S first decode *x*(*t*) with the received signal-to-interference-plus-noise ratio (SINR), denoted by $\gamma_{x}$ and then *c*(*t*) with the received signal-to-noise ratio (SNR) $\gamma_{c}$ by adopting the successive interference cancelation (SIC) technique [50,51]. As a result, the end-to-end (e2e) received SNR at source must hold the following condition

$$\gamma_{e2e}=\min\{\gamma_{x},\gamma_{c}\}$$ (2)

To truly enhance the effectiveness of symbiotic IoT communication in short-packet uplink transmission, it’s crucial to address the unique challenges presented by both the direct user-source link and the cascaded BD-source link. By implementing three strategic diversity techniques at the source, we can significantly boost reliability and performance across various signal directions. This approach not only tackles existing issues but also optimizes overall system efficiency, making it an essential step forward in advancing IoT technology.

- **SC-RAN (Selective Combining - Random Selection)**: By employing SC for the user-source link in this approach, we ensure the selection of the strongest signal path for decoding *x*(*t*). This strategy significantly minimizes the impact of channel variations, making it a highly effective solution. At the same time, RAN is strategically employed for the BD-source link to effectively select the random arriving signal from the cascaded channel $h_{2}h_{3}$ for *c*(*t*).
- **SC-MRC (Selective Combining - Maximal Ratio Combining)**: In this strategy, MRC is used for the BD-source link to smoothly blend signals coming from the cascaded channel $h_{2}h_{3}$, which helps boost the SNR for *c*(*t*). This approach keeps things simple and works efficiently by cutting down on complexity for the direct user-source link, all while making the weaker cascaded BD-source link more reliable.
- **Full-MRC (Full-Maximal Ratio Combining)**: This strategy decisively applies MRC to both user-source and BD-source links. By effectively integrating all available signal paths for *x*(*t*) and *c*(*t*), Full-MRC maximizes received signal strength while minimizing interference and noise throughout the system. It undeniably offers superior reliability and decoding performance over SC-RAN and SC-MRC. However, it demands higher computational complexity and resource allocation, making it ideal only for situations where energy and processing resources are not limited.

These diversity techniques are essential for tackling the performance degradation resulting from the cascaded channel in symbiotic communication. Utilizing SC-RAN, SC-MRC or Full-MRC is non-negotiable if the system is to meet the strict reliability and low-latency demands of finite blocklength communication. This approach guarantees successful decoding of both *x*(*t*) and *c*(*t*), thereby securing effective end-to-end communication. From the strategies outlined above, it is clear that the e2e received SNR at the source over M-antennas can be confidently asserted as

$$\gamma_{\text{e2e}}=\{\begin{array}{c} \min\left(\frac{\Psi X}{\beta \Psi Y+1},\beta \Psi Y\right),\;\;\text{SC - RAN}\\ \min\left(\frac{\Psi X}{\beta \Psi Z+1},\beta \Psi Z\right),\;\text{SC - MRC}\\ \min\left(\frac{\Psi T}{\beta \Psi Z+1},\beta \Psi Z\right),\;\text{full--MRC} \end{array}$$ (3)

where $X\overset{\Delta }{=}\underset{m=1,...,M}{\underbrace{\max}}{\left|h_{1}^{m}\right|}^{2},Y\overset{\Delta }{=}{\left|h_{2}\right|}^{2}{\left|h_{3}\right|}^{2},Z={\left|h_{2}\right|}^{2}\sum_{m=1}^{M}{\left|h_{3}^{m}\right|}^{2},T=\sum_{m=1}^{M}{\left|h_{1}^{m}\right|}^{2}$, and $\Psi =\frac{P_{\text{U}}}{N_{0}}$ is the average SNR.

## 3 Channel statistics and preliminary of finite blocklength

In this section, we conduct a thorough examination of the proposed system’s performance. We develop approximation form expressions for the block error ratio of the three strategies outlined in Eq (3) to facilitate a detailed analysis of each strategy. This approach enables us to propose an appropriate method for enhancing the system’s quality.

### 3.1 Channel statistics

Based on the description of the proposed system in Sect 2, the channel gain ${\left|h_{i}\right|}^{2}\left\{i\in \left(1,2,3\right)\right\}$ will follow the exponential distribution. Hence, its cumulative distribution function (CDF) and probability density function (PDF) can be given as, respectively.

$$\begin{array}{c} F_{{\left|h_{i}\right|}^{2}}(x)=1-\exp\left(-\lambda_{i}x\right),\\ f_{{\left|h_{i}\right|}^{2}}(x)=\lambda_{i}\exp\left(-\lambda_{i}x\right), \end{array}$$ (4)

where $\lambda_{i}$ is the mean of ${\left|h_{i}\right|}^{2}$. To take into account the impacts of the path-loss model, $\lambda_{i}$ can be expressed by $\lambda_{i}=(d_{i}{)}^{\chi }$, where *d*_*i*_ is the distance related to ${\left|h_{i}\right|}^{2}$ while χ is the path-loss exponent.

### 3.2 Preliminary of finite blocklength

For a given *N*, i.e., the number of the information bit transmitted to S and *L*, i.e., the block-length (packet length) or the number of channel use, the e2e average BLER for decoding the signal *x*(*t*) can be given as [23,24]

$$\Xi_{\text{S}}=Q\left(\frac{C\left(\gamma_{\text{e2e}}-r\right)}{\sqrt{\frac{U\left(\gamma_{\text{e2e}}\right)}{L}}}\right),$$ (5)

where $Q(q)=\int_{q}^{\infty }\frac{1}{\sqrt{2\pi }}e^{-y^{2}/2}dy$, $C(q)=\log_{2}\left(1+q\right)$ are the Gaussian Q-function, the Shannon capacity, respectively and $U(q)=\log_{2}(e{)}^{2}\left[1-1/(1+q)\right]$ is the channel dispersion, while $\frac{r\overset{\Delta }{=}}{=N/L.}$

## 4 BLER analysis

Based on [52, Appendix A], the approximation expression of BLER can be claimed by

$$\Upsilon_{\text{S}}(\gamma_{\text{e2e}})=\left\{ \begin{array}{c} 1,\;\;\;\;\;\;\;\;\;\;\;\;\;\;\;\;\;\;\;\;\;\;\;\;\;\;\;\;\;\;\;\;\;\;\;\;\;\;\;\;\;\;\gamma_{\text{e2e}}\leq \upsilon \\ 0,\;\;\;\;\;\;\;\;\;\;\;\;\;\;\;\;\;\;\;\;\;\;\;\;\;\;\;\;\;\;\;\;\;\;\;\;\;\;\;\;\;\gamma_{\text{e2e}}\geq u\\ \frac{1}{2}-\Theta \left(\gamma_{\text{e2e}}-\tau \right),\;\;\upsilon \leq \gamma_{\text{e2e}}\leq u \end{array}\right.$$ (6)

where $\Theta ={\left\{\frac{2\pi \left(2^{2r}-1\right)}{L}\right\}}^{-\frac{1}{2}}$, $\tau =2^{r}-1,\upsilon =\tau -1/\left(2\Theta \right)$, and u=τ+1/(2Θ).

Finally, by substituting (6) into (5), the e2e average BLER can be obtained as

$$\Xi_{\text{S}}\approx \int_{0}^{\infty }\Upsilon_{\text{S}}(\gamma_{\text{e2e}})f_{\gamma_{\text{e2e}}}(x)dx\approx \Theta \int_{\upsilon }^{u}F_{\gamma_{\text{e2e}}}(x)dx$$ (7)

By observing (7), to obtain BLER expressions of the three strategies mentioned in (3), we will try to compute the CDF of the e2e received SNR at S for these scenarios in closed-form expressions. First of all, the CDF of *X* as in [53], the CDF and PDF of *T* as in [54] can be expressed by, respectively.

$$\begin{array}{c} F_{X}(x)=1-\sum_{m\;\;=1}^{M}\left(\begin{array}{c} M\\ m \end{array}\right){\left(-1\right)}^{m-1}\exp\left(-m\lambda_{1}x\right),\\ F_{T}(t)=\frac{1}{\Gamma \left(\text{M}\right)}\gamma \left(\text{M},\lambda_{\text{1}}x\right),\\ f_{T}(t)=\frac{{\left(\lambda_{1}\right)}^{M}}{\Gamma \left(\text{M}\right)}t^{M-1}\exp(-\lambda_{1}t), \end{array}$$ (8)

where Γ(.) is the Gamma function and γ(a,x) is the incomplete gamma function.

### 4.1 SC-RAN

In this scenario, from (3), the CDF of $\gamma_{\text{e2e}}$ can be derived as

$$\begin{array}{c} F_{\gamma_{\text{e2e}}}^{\text{SC - RAN}}(x)=\mathrm{Pr}\left(\min\left(\frac{\Psi X}{\beta \Psi Y+1},\beta \Psi Y\right)<x\right)\\ =1-\mathrm{Pr}\left(\frac{\Psi X}{\beta \Psi Y+1}\geq x,\beta \Psi Y\geq x\right)\\ =1-\int_{\frac{x}{\beta \Psi }}^{+\infty }\left\{1-F_{X}\left(x\beta y+\frac{x}{\Psi }\right)\right\}\times f_{Y}\left(y\right)dy. \end{array}$$ (9)

From (9), $f_{Y}(y)=\frac{\partial F_{Y}(y)}{\partial y}$ can be given by [55]

$$f_{Y}(y)=2\lambda_{\text{2}}\lambda_{\text{3}}\times K_{0}\left(2\sqrt{\lambda_{\text{2}}\lambda_{\text{3}}y}\right),$$ (10)

where $K_{v}\left(.\right)$ is the modified Bessel function of second kind with $v^{\text{th}}$ order.

By applying (8) and (10), Eq (9) can be rewritten by

$$\begin{array}{c} F_{\gamma_{\text{e2e}}}^{\text{SC - RAN}}(x)=1-2\sum_{m\;\;=1}^{M}\left(\begin{array}{c} M\\ m \end{array}\right){\left(-1\right)}^{m-1}\lambda_{2}\lambda_{3}\exp\left(-\frac{m\lambda_{1}x}{\Psi }\right)\\ \times \underset{\Delta_{1}}{\underbrace{\int_{\frac{x}{\beta \Psi }}^{+\infty }\exp\left(-m\lambda_{1}\beta xy\right)\times K_{0}\left(2\sqrt{\lambda_{2}\lambda_{3}y}\right)dy}}. \end{array}$$ (11)

By setting $t=\frac{y\beta \Psi }{x}$, the $\Delta_{1}$ in (11) is formulated as

$$\Delta_{1}=\frac{x}{\beta \Psi }\int_{1}^{+\infty }\exp\left(-\frac{m\lambda_{1}x^{2}t}{\Psi }\right)\times K_{0}\left(2\sqrt{\frac{\lambda_{2}\lambda_{3}xt}{\beta \Psi }}\right)dt.$$ (12)

Next, by using Taylor series for $\exp\left(-\frac{m\lambda_{1}x^{2}t}{\Psi }\right)=\sum_{n\geq 0}\frac{\left(-\frac{m\lambda_{1}x^{2}t}{\Psi }\right)}{n!}$ and then substituting into (12), we have:

$$\Delta_{1}=\frac{x}{\beta \Psi }\sum_{n\geq 0}\frac{{\left(-\frac{m\lambda_{1}x^{2}}{\Psi }\right)}^{n}}{n!}\int_{1}^{+\infty }t^{n}K_{0}\left(2\sqrt{\frac{\lambda_{2}\lambda_{3}xt}{\beta \Psi }}\right)dt.$$ (13)

Based on [56, eq:6.592.4], $\Delta_{1}$ can be thus reformulated by

$$\Delta_{1}=\frac{x}{2\beta \Psi }\sum_{n\geq 0}\frac{\left(-\frac{m\lambda_{1}x\beta }{\lambda_{2}\lambda_{3}}\right)}{n!}\times G_{1,3}^{3,0}\left(\frac{\lambda_{2}\lambda_{3}x}{\beta \Psi }|\begin{array}{c} 0\\ -1,n,n \end{array}\right),$$ (14)

where $\;G_{p,q}^{m,n}\left({x|}_{b_{1},\;...,\;b_{q}}^{a_{1},\;...,\;a_{p}}\right)$ is the Meijer function.

Next, we can obtain the closed-form expression of $F_{\gamma_{\text{e2e}}}^{\text{SC - RAN}}(x)$ by substituting (14) into (11). Then, substituting the obtaining $F_{\gamma_{\text{e2e}}}^{\text{SC - RAN}}(x)$ in (11) into (7), we claim:

$$\begin{array}{c} \Xi_{\text{S}}^{\text{SC - RAN}}=1-\frac{\Theta \lambda_{2}\lambda_{3}}{\beta \Psi }\sum_{m=1}^{M}\sum_{n\geq 0}\left(\begin{array}{c} M\\ m \end{array}\right)\frac{{\left(-1\right)}^{m-1}{\left(-\frac{m\lambda_{1}\beta }{\lambda_{2}\lambda_{3}}\right)}^{n}}{n!}\\ \times \int_{\upsilon }^{u}x^{n+1}\exp\left(-\frac{m\lambda_{1}x}{\Psi }\right)\times G_{1,3}^{3,0}\left(\frac{\lambda_{2}\lambda_{3}x}{\beta \Psi }|\begin{array}{c} 0\\ -1,n,n \end{array}\right)dx. \end{array}$$ (15)

Unfortunately, the integral in (15) is a tough task to figure out a closed-form expression. Therefore, by applying the Gaussian-Chebyshev quadrature as in [57], the e2e average BLER in this strategy can be given as

$$\begin{array}{c} \Xi_{\text{S}}^{\text{SC - RAN}}\approx 1-\frac{\pi \lambda_{2}\lambda_{3}}{2K\beta \Psi }\sum_{m=1}^{M}\sum_{n\geq 0}\sum_{k=1}^{K}\sqrt{1-\phi_{k}^{2}}\left(\begin{array}{c} M\\ m \end{array}\right)\frac{{\left(-1\right)}^{m-1}{\left(-\frac{m\lambda_{1}\beta }{\lambda_{2}\lambda_{3}}\right)}^{n}}{n!}\\ \times {\left(\frac{\phi_{k}}{2\Theta }+\tau \right)}^{n+1}\exp\left(-\frac{m\lambda_{1}\left[\frac{\phi_{k}}{2\Theta }+\tau \right]}{\Psi }\right)\times G_{1,3}^{3,0}\left(\frac{\lambda_{2}\lambda_{3}\left[\frac{\phi_{k}}{2\Theta }+\tau \right]}{\beta \Psi }|\begin{array}{c} 0\\ -1,n,n \end{array}\right), \end{array}$$ (16)

where $\phi_{k}=\cos\left(\frac{2k-1}{K}\pi \right)$.

### 4.2 SC-MRC

Based on (3), the CDf of $\gamma_{\text{e2e}}$ can be achieved as

$$F_{\gamma_{\text{e2e}}}^{\text{SC - MRC}}(x)=1-\int_{\frac{x}{\beta \Psi }}^{+\infty }\left\{1-F_{X}\left(x\beta z+\frac{x}{\Psi }\right)\right\}\times f_{Z}(z)dz.$$ (17)

In order to get the closed-form expression for (17). Firstly, we compute the CDF of *Z* random variable (RV), which *Z* is the product of ${\left|h_{2}\right|}^{2}$ and $\sum_{m=1}^{M}{\left|h_{3}^{m}\right|}^{2}$ as following:

$$\begin{array}{c} F_{Z}(z)=\mathrm{Pr}\left(Z<z\right)=\mathrm{Pr}\left({\left|h_{2}\right|}^{2}\sum_{m=1}^{M}{\left|h_{3}^{m}\right|}^{2}<z\right)\\ =\int_{0}^{+\infty }F_{{\left|h_{2}\right|}^{2}}\left(\frac{z}{t}\right)\times f_{\sum_{m=1}^{M}{\left|h_{3}^{m}\right|}^{2}}(t)dt. \end{array}$$ (18)

By applying (3) and (8), Eq (18) is taken by

$$F_{Z}(z)=1-\frac{\lambda_{3}^{M}}{\Gamma \left(M\right)}\int_{0}^{+\infty }t^{M-1}\exp\left(-\frac{\lambda_{2}z}{t}-\lambda_{3}t\right)dt,$$ (19)

where Γ(.) is the Gamma function.

Later, with the help of [56, eq:3.471.9], we gather as follows

$$F_{Z}(z)=1-\frac{2{\left(\lambda_{2}\lambda_{3}z\right)}^{\frac{M}{2}}}{\Gamma \left(M\right)}\times K_{M}\left(2\sqrt{\lambda_{2}\lambda_{3}z}\right).$$ (20)

From the above result, the PDF of *Z* can be attained by using $\frac{d}{dx}\left(x^{v}K_{v}(x)\right)=-x^{v}K_{v-1}(x)$ as below

$$f_{Z}(z)=\frac{\partial F_{Z}(z)}{\partial z}=\frac{2{\left(\lambda_{2}\lambda_{3}\right)}^{\frac{M+1}{2}}z^{\frac{M-1}{2}}}{\Gamma \left(M\right)}\times K_{M-1}\left(2\sqrt{\lambda_{2}\lambda_{3}z}\right).$$ (21)

Next, by plugging (8) and (21) into (17), we receive:

$$\begin{array}{c} F_{\gamma_{\text{e2e}}}^{\text{SC - MRC}}(x)=1-2\sum_{m=1}^{M}\left(\begin{array}{c} M\\ m \end{array}\right)\frac{{\left(-1\right)}^{m-1}{\left(\lambda_{2}\lambda_{3}\right)}^{\frac{M+1}{2}}}{\Gamma \left(M\right)}\times \exp\left(-\frac{m\lambda_{1}x}{\Psi }\right)\\ \times \int_{\frac{x}{\beta \Psi }}^{+\infty }z^{\frac{M-1}{2}}\exp\left(-m\lambda_{1}x\beta z\right)\times K_{M-1}\left(2\sqrt{\lambda_{2}\lambda_{3}z}\right)dz. \end{array}$$ (22)

By using the same approach as SC-RAN case, with applying the Taylor series for $\exp\left(-\frac{m\lambda_{1}x^{2}t}{\Psi }\right)$ and then adopting [56, eq:6.592.4], the closed-form expression of $F_{\gamma_{\text{e2e}}}^{\text{SC - MRC}}$ can be procured by

$$\begin{array}{c} F_{\gamma_{\text{e2e}}}^{\text{SC - MRC}}(x)=1-\sum_{m=1}^{M}\sum_{n\geq 0}\left(\begin{array}{c} M\\ m \end{array}\right)\frac{{\left(-1\right)}^{m-1}{\left(\lambda_{2}\lambda_{3}\right)}^{-n+1}{\left(-m\lambda_{1}\right)}^{n}}{\Gamma \left(M\right){\left(\beta \Psi \right)}^{1-n}\left(n!\right)}\\ \times x^{n+1}\exp\left(-\frac{m\lambda_{1}x}{\Psi }\right)\times G_{1,3}^{3,0}\left(\frac{\lambda_{2}\lambda_{3}x}{\beta \Psi }|\begin{array}{c} 0\\ -1,\frac{M+1}{2},\frac{-M+3}{2} \end{array}\right). \end{array}$$ (23)

Finally, by replacing (23) into (7) and afterwards by utilizing Gaussian-Chebyshev quadrature as in SC-RAN case, we achieve:

$$\begin{array}{c} \Xi_{\text{S}}^{\text{SC - MRC}}\approx 1-\frac{\pi }{2K}\sum_{m=1}^{M}\sum_{n\geq 0}\sum_{k=1}^{K}\left(\begin{array}{c} M\\ m \end{array}\right)\frac{{\left(-1\right)}^{m-1}{\left(\lambda_{2}\lambda_{3}\right)}^{-n+1}{\left(-m\lambda_{1}\right)}^{n}}{\Gamma \left(M\right){\left(\beta \Psi \right)}^{1-n}\left(n!\right)}\\ \times {\left(\frac{\phi_{k}}{2\Theta }+\tau \right)}^{n+1}\exp\left(-\frac{m\lambda_{1}\left[\frac{\phi_{k}}{2\Theta }+\tau \right]}{\Psi }\right)\times G_{1,3}^{3,0}\left(\frac{\lambda_{2}\lambda_{3}\left[\frac{\phi_{k}}{2\Theta }+\tau \right]}{\beta \Psi }|\begin{array}{c} 0\\ -1,\frac{M+1}{2},\frac{-M+3}{2} \end{array}\right). \end{array}$$ (24)

### 4.3 Full MRC

In this final strategy, we delve into the MRC diversity technique, which is crucial for optimizing both U-S and BD-S links. By understanding and implementing this approach, we can significantly enhance link performance and reliability. This technique is not just an option; it’s a strategic necessity for achieving superior connectivity outcomes. From (3), the CDF of full-MRC scenario can be found as

$$\begin{array}{c} F_{\gamma_{\text{e2e}}}^{\text{full--MRC}}(x)\approx 1-\mathrm{Pr}\left(\frac{T}{\beta Z}\geq x,\beta \Psi Z\geq x\right)\\ =1-\int_{\frac{x}{\beta \Psi }}^{+\infty }\left\{1-F_{T}\left(x\beta z\right)\right\}\times f_{Z}(z)dz. \end{array}$$ (25)

By alternating (8) and (21) into (25), we get:

$$F_{\gamma_{\text{e2e}}}^{\text{full--MRC}}(x)=1-\int_{\frac{x}{\beta \Psi }}^{+\infty }\left[\begin{array}{c} \left\{1-\frac{1}{\Gamma \left(M\right)}\gamma \left(M,\lambda_{1}x\beta z\right)\right\}\\ \times \frac{2{\left(\lambda_{2}\lambda_{3}\right)}^{\frac{M+1}{2}}z^{\frac{M-1}{2}}}{\Gamma \left(M\right)}\times K_{M-1}\left(2\sqrt{\lambda_{2}\lambda_{3}z}\right) \end{array}\right]dz.$$ (26)

Next, we perform the series expansion for the incomplete gamma function by referring [56, eq:8.352.6] as following:

$$\gamma \left(M,\lambda_{1}x\beta z\right)=\Gamma \left(M\right)\left[1-\exp\left(-\lambda_{1}x\beta z\right)\sum_{p=0}^{M-1}\frac{{\left(\lambda_{1}x\beta z\right)}^{p}}{p!}\right].$$ (27)

By substituting (27) into (26) and after doing some algebra, we obtain:

$$\begin{array}{c} F_{\gamma_{\text{e2e}}}^{\text{full--MRC}}(x)=1-2\sum_{p=0}^{M-1}\frac{{\left(\lambda_{2}\lambda_{3}\right)}^{\frac{M+1}{2}}{\left(\lambda_{1}x\beta \right)}^{p}}{p!\Gamma \left(M\right)}\\ \times \underset{\Delta_{3}}{\underbrace{\int_{\frac{x}{\beta \Psi }}^{+\infty }z^{\frac{2p+M-1}{2}}\exp\left(-\lambda_{1}x\beta z\right)\times K_{M-1}\left(2\sqrt{\lambda_{2}\lambda_{3}z}\right)dz}}. \end{array}$$ (28)

After changing variable $t=\frac{z\beta \Psi }{x}$ and using Taylor series as the same in (12), $\Delta_{3}$ in (28) can be achieved as

$$\Delta_{3}={\left(\frac{x}{\beta \Psi }\right)}^{\frac{2p+M+1}{2}}\sum_{n\geq 0}\frac{\left(-\frac{\lambda_{1}x^{2}}{\Psi }\right)}{n!}\int_{1}^{+\infty }t^{\frac{2n+2p+M-1}{2}}\times K_{M-1}\left(2\sqrt{\frac{\lambda_{2}\lambda_{3}xt}{\beta \Psi }}\right)dt.$$ (29)

Recalling [56, eq:6.592.4], $\Delta_{3}$ is thus gathered by

$$\Delta_{3}=\sum_{n\geq 0}\frac{{\left(-\frac{\lambda_{1}}{\Psi }\right)}^{n}{\left(\lambda_{2}\lambda_{3}\right)}^{-n-p-\frac{M-1}{2}}}{2\times \left(n!\right){\left(\beta \Psi \right)}^{1-n}}\times x^{n+1}\times G_{1,3}^{3,0}\left(\frac{\lambda_{2}\lambda_{3}x}{\beta \Psi }|\begin{array}{c} 0\\ -1,m+p+M-1,p+n \end{array}\right).$$ (30)

Subsequently, by plugging (30) into (28), the closed-form expression of $F_{\gamma_{\text{e2e}}}^{\text{full--MRC}}(x)$ can be derived as

$$\begin{array}{c} F_{\gamma_{\text{e2e}}}^{\text{full--MRC}}(x)=1-\sum_{p=0}^{M-1}\sum_{n\geq 0}\frac{{\left(-1\right)}^{n}{\left(\lambda_{1}\right)}^{p+n}\beta^{p+n-1}{\left(\lambda_{2}\lambda_{3}\right)}^{-n-p+1}}{n!p!\Gamma \left(M\right)\Psi }\\ \times x^{p+n+1}\times G_{1,3}^{3,0}\left(\frac{\lambda_{2}\lambda_{3}x}{\beta \Psi }|\begin{array}{c} 0\\ -1,p+n+M-1,p+n \end{array}\right). \end{array}$$ (31)

In the end, by surrogating (31) into (7) and then applying Gaussian-Chebyshev quadrature again, the e2e average BLER expression of full MRC scenario can be acquired by

$$\begin{array}{c} \Xi_{\text{S}}^{\text{full--MRC}}\approx 1-\frac{\pi }{2K}\sum_{k=1}^{K}\sum_{p=0}^{M-1}\sum_{n\geq 0}\frac{{\left(-1\right)}^{n}{\left(\lambda_{1}\right)}^{p+n}\beta^{p+n-1}{\left(\lambda_{2}\lambda_{3}\right)}^{-n-p+1}}{n!p!\Gamma \left(M\right)\Psi }\\ \times {\left(\frac{\phi_{k}}{2\Theta }+\tau \right)}^{p+n+1}\times G_{1,3}^{3,0}\left(\frac{\lambda_{2}\lambda_{3}\left[\frac{\phi_{k}}{2\Theta }+\tau \right]}{\beta \Psi }|\begin{array}{c} 0\\ -1,p+n+M-1,p+n \end{array}\right). \end{array}$$ (32)

## 5 Numerical results

In this section, we rigorously evaluate our theoretical findings on the performance of e2e average BLER through a comprehensive numerical assessment. We establish the mean of random variables (RVs) ${\left|h_{1}\right|}^{2}$, ${\left|h_{2}\right|}^{2}$, ${\left|h_{3}\right|}^{2}$ as $\lambda_{1}$=0.5, $\lambda_{2}$=1, and $\lambda_{3}$=2. The results achieved from Monte Carlo simulations [58–60] are averaged over 10^6^ independent trials. In the subsequent figures, we denote that the dashed lines represent the Monte Carlo simulation, and the others are analytical computations.

**Fig 2:**
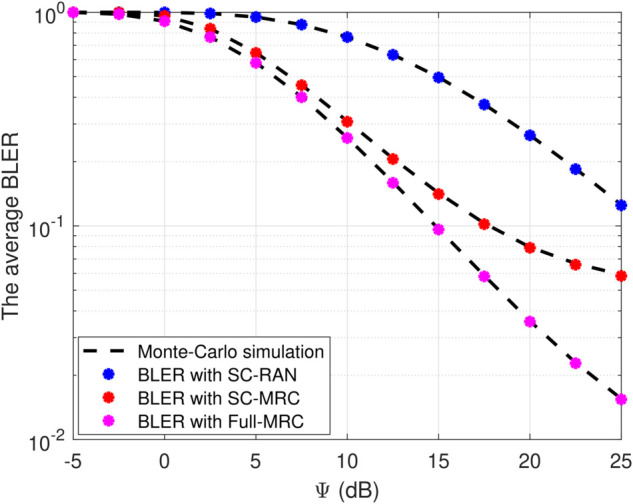
BLER versus the average transmit power Ψ with M=5, β=0.25, N=200, L=150.

Fig 2 compellingly illustrates the impact of different reception methods on the average BLER as the average SNR Ψ varies. The Full-MRC design stands out remarkably, demonstrating a significant reduction in BLER with increasing SNR, which clearly highlights its superior performance. This evidence strongly supports Full-MRC’s ability to effectively harness geographical diversity and optimize end-to-end reliability. Meanwhile, SC-MRC offers a balanced approach by gradually improving efficiency while maintaining manageable complexity. In contrast, SC-RAN exhibits the highest BLER due to its limited use of diversity through selective combining only. The precise alignment between analytical curves and Monte Carlo simulations further validates the accuracy of our derived closed-form formulas. These findings convincingly establish Full-MRC as the most robust system, making it an ideal choice for URLLC scenarios where reliability is paramount.

**Fig 3:**
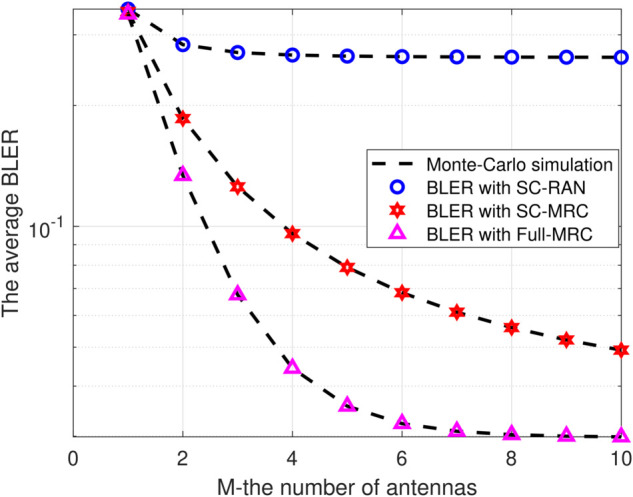
BLER versus the number of antennas M with Ψ=20(dB), β=0.25, N=200, L=150.

Fig 3 clearly demonstrates that as M increases from 1 to 10, the average BLER decreases across the scenarios of SC-RAN, SC-MRC, and Full-MRC because of enhancing the SNR, channel capacity or energy efficiency. Moreover, this trend is due to the enhanced diversity gain at the receiver with more antennas. Notably, Fig 3 also shows that the Full-MRC method outperforms the others. However, it’s crucial to consider that increasing antenna numbers can complicate hardware implementation. Therefore, selecting an optimal M is essential for ensuring system reliability while balancing complexity and performance effectively.

**Fig 4:**
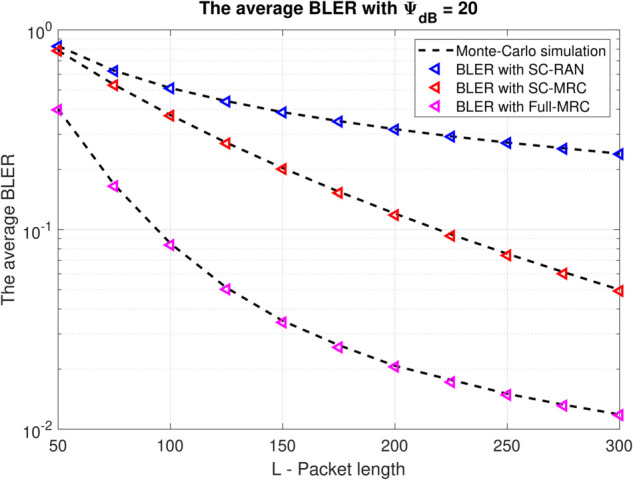
BLER versus the number of packet length L with Ψ=20(dB), β=0.25, N=100, M=4.

Fig 4 depicts the impact of packet length L on the average BLER for three methods SC-RAN, SC-MRC and Full-MRC schemes. As expected from finite blocklength theory [cf. (5)], increasing the packet length significantly reduces the BLER due to improved coding redundancy and error correction capability. This is clearly shown by the downward trend of all curves as L increases. Furthermore, the Full-MRC scheme consistently outperforms SC-MRC and SC-RAN across all scenarios, which aligns with the earlier analysis and reinforces the effectiveness of full diversity combining. The performance gap between SC-MRC and SC-RAN or SC-RAN and Full-MRC is especially notable in the moderate and high L regime, where systems must balance delay constraints and reliability, such as in URLLC applications.

**Fig 5:**
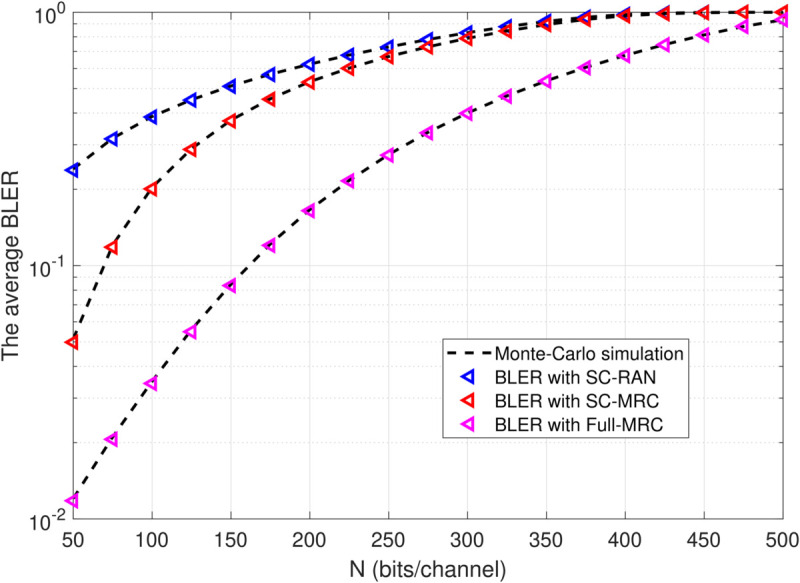
BLER versus the number of the information bit N with Ψ=20(dB), β=0.25, L=150, M=4.

Fig 5 presents the average BLER as a function of the number of information bits, N, across three scenarios. As N increases, the BLER of our proposed system also increases. This occurs because transmitting more bits within a block increases the likelihood that at least one bit will be corrupted by noise, interference, or other channel impairments, which degrades decoding reliability. This trend illustrates the inherent trade-off in short-packet communications, where larger blocklengths may support higher data rates but come at the cost of higher BLER.

**Fig 6:**
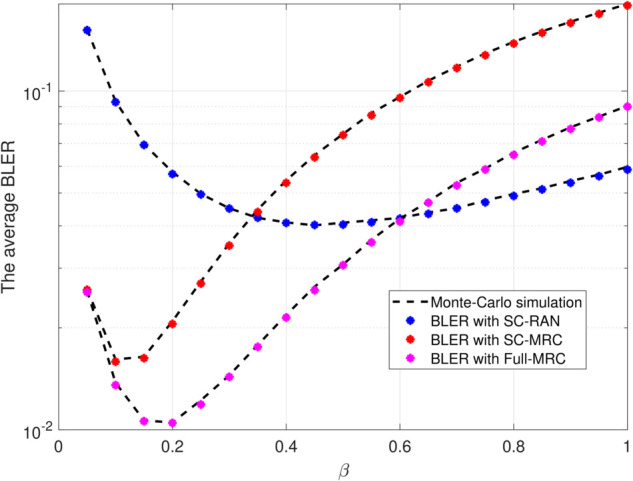
BLER versus the reflection coefficient (*β*) with Ψ=20(dB), N=100, L=300, M=3.

Fig 6 illustrates that the average BLER first decreases as *β* increases, thanks to stronger backscattered signals, but then rises again when *β* becomes too large because the backscatter device consumes excessive energy for reflection, leading to a risk of energy outage. This concave trend indicates the existence of an optimal *β* that balances reflection strength and energy storage capability. Specifically, SC-RAN achieves its minimum BLER around β=0.45, SC-MRC around β=0.1, and Full-MRC around β=0.2, highlighting the importance of selecting an appropriate *β* for each scenario. Across the entire range of *β* values, Full-MRC consistently attains the lowest BLER, followed by SC-MRC, while SC-RAN exhibits the highest BLER and thus the lowest reliability.

**Fig 7:**
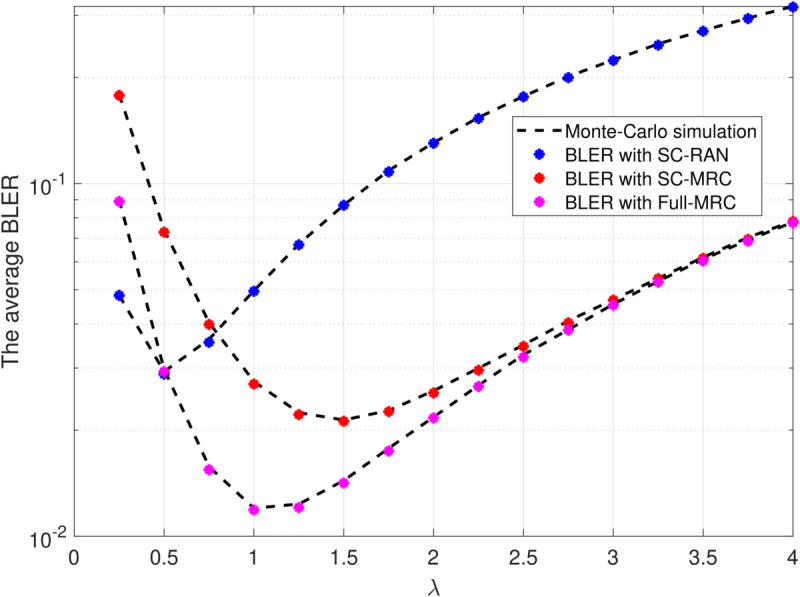
BLER versus $\lambda =\lambda_{1}=\lambda_{2}=\lambda_{3}$ with Ψ=20(dB), β=0.25, L=300, N=100, M=3.

Fig 7 reveals a U-shaped relationship between the average BLER and the common channel parameter $\lambda =\lambda_{1}=\lambda_{2}=\lambda_{3}$, where each $\lambda_{i}$ represents the mean of the squared channel gain $|h_{i}{|}^{2}$. As *λ* increases from very small values, the average channel power improves and the BLER drops sharply. Notably, for λ<0.5, the SC-RAN curve lies below those of SC-MRC and Full-MRC, showing that SC-RAN is advantageous under extremely weak channel conditions. As *λ* grows further, however, SC-RAN quickly loses this advantage, while SC-MRC and especially Full-MRC achieve lower BLER. Around moderate *λ*, each scheme reaches its own minimum (SC-RAN = 0.5, SC-MRC = 1.0, Full-MRC = 1.5). At large *λ*, the BLER of all three schemes rises again, but Full-MRC consistently remains the most reliable.

## 6 Conclusion

This work presents a unified framework that integrates uplink D2D communication, backscatter transmission, and short-packet communication to address the critical requirements of URLLC in emerging wireless systems. We derive approximation-form expressions for the average BLER under three diversity strategies—SC-RAN, SC-MRC, and Full-MRC—using the Gaussian-Chebyshev quadrature method and validate them through extensive Monte Carlo simulations. The results unequivocally demonstrate that Full-MRC delivers superior reliability, positioning it as a promising solution for mission-critical IoT and energy-constrained applications. Moreover, the insights gained from this analysis play a pivotal role in optimizing key parameter choices within the proposed system architecture. Beyond theoretical contributions, this study also provides practical design insights for low-latency wireless systems. In addition, this study points out the complexity–practicality trade-off in diversity combining. While Full-MRC provides the highest reliability, it also involves greater computational and hardware requirements, which may limit its practicality in IoT deployments. In contrast, SC-MRC offers a promising balance between performance and complexity, making it more attractive for resource-constrained scenarios. A more quantitative evaluation of computational complexity and hardware overhead will be considered in our future work. Future research will focus on addressing imperfect channel state information, optimizing reflection coefficients, and extending the analysis to multi-backscatter scenarios with adaptive combining techniques. While the current work considers a single backscatter device for analytical tractability, extending the framework to multiple backscatter devices is highly relevant for dense IoT deployments. Such an extension will involve new challenges, including interference coupling and resource allocation among devices, and will therefore require more elaborate modeling and simulation. We consider this an important direction and have explicitly included it as part of our future research agenda. These extensions are expected to further enhance the robustness and scalability of next-generation symbiotic networks.
